# Structural insight into the mechanism of synergistic autoinhibition of SAD kinases

**DOI:** 10.1038/ncomms9953

**Published:** 2015-12-02

**Authors:** Jing-Xiang Wu, Yun-Sheng Cheng, Jue Wang, Lei Chen, Mei Ding, Jia-Wei Wu

**Affiliations:** 1 MOE Key Laboratory for Protein Science and Tsinghua-Peking Center for Life Sciences, School of Life Sciences, Tsinghua University, Beijing 100084, China; 2 State Key Laboratory of Molecular Developmental Biology, Institute of Genetics and Developmental Biology, Chinese Academy of Sciences, Beijing 100101, China

## Abstract

The SAD/BRSK kinases participate in various important life processes, including neural development, cell cycle and energy metabolism. Like other members of the AMPK family, SAD contains an N-terminal kinase domain followed by the characteristic UBA and KA1 domains. Here we identify a unique autoinhibitory sequence (AIS) in SAD kinases, which exerts autoregulation in cooperation with UBA. Structural studies of mouse SAD-A revealed that UBA binds to the kinase domain in a distinct mode and, more importantly, AIS nestles specifically into the KD-UBA junction. The cooperative action of AIS and UBA results in an ‘αC-out' inactive kinase, which is conserved across species and essential for presynaptic vesicle clustering in *C. elegans*. In addition, the AIS, along with the KA1 domain, is indispensable for phospholipid binding. Taken together, these data suggest a model for synergistic autoinhibition and membrane activation of SAD kinases.

## Abstract (web summary)

The SAD kinases contain a UBA domain that binds to the kinase domain and has a role in autoinhibition and allosteric activation of the AMPK homoenzyme. Here, the authors identify an autoinhibitory sequence in SAD and show that the UBA domain synergistically functions as an autoinhibitory domain.

## Graphical abstract

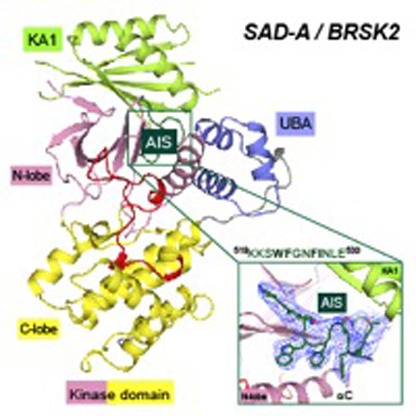

The SAD (synapses of amphids defective) kinases, conserved across a wide range of species, play important roles in multiple phases of axonal development and function. The founder member *Caenorhabditis elegans* SAD-1 was identified from a screen for mutations affecting presynaptic vesicle clustering at active zones, and SAD-1 was demonstrated to regulate axon termination and neuronal polarity^1^. Later, mammalian SAD-A and SAD-B (also referred to as BRSK2 and BRSK1, respectively) have emerged as essential regulators of early axon–dendrite polarization and axonal arborization^2^,^3^,^4^,^5^,^6^. The SAD kinases have also been reported to localize to synaptic sites and promote the maturation of nerve terminals or modulate neurotransmitter release in adults^7^,^8^. In addition to the significant functions in neurons, mammalian SAD-B and the yeast orthologs Cdr2 and Hsl1 are involved in cell cycle by regulating mitotic entry and centrosome biogenesis^9^,^10^,^11^,^12^,^13^. Recently, evidence had unravelled striking roles of the SAD-A in glucose-stimulated insulin secretion, GLP-1 response and mTORC1 signalling in islet β cells^14^,^15^,^16^. The important progresses in understanding the contribution of SAD kinases in diverse biological processes renders them attractive drug targets for treating neurological diseases, cancer and metabolic disorders. Like other kinases, functions of the SAD kinases depend on their catalytic activities. The activated SAD kinases can phosphorylate various downstream effector proteins including Tau, RIM1, Wee1, Cdc25, γ-tubulin and PAK1, and thereby regulate diverse biological processes^2^,^3^,^7^,^12^,^14^,^17^. However, little is known about the regulatory mechanisms of SAD activity.

In human genome, BRSK1/SAD-B and BRSK2/SAD-A are closely related to AMPK-α1/2 subunits and 10 other kinases (MARK1/2/3/4, SIK1/2/3, NUAK1/2 and MELK), and these kinases constitute a unique branch of the human kinome tree, the AMPK family^18^. The principal member AMPK, a heterotrimer consisting of the catalytic α- and regulatory β-, γ-subunits, is a key regulator of cellular and whole-body energy homoeostasis^19^,^20^. Recently, AMPK activation under metabolic stress have been reported to suppress axon initiation and neuronal polarization, and Aβ-induced activation of AMPK can trigger dendritic spine loss^21^,^22^,^23^. The MARK/Par-1 subfamily regulates neuronal differentiation and migration, as well as various biological processes including cell polarity, cell cycle control and glucose metabolism^24^,^25^. Despite the diverse functions, the AMPK family members display similar structural organization, with an N-terminal kinase domain followed by a non-catalytic region containing an ubiquitin associated (UBA) domain and, in some cases, a kinase associated (KA1) domain. Their catalytic activities are generally coupled to phosphorylation of a conserved threonine in the kinase domain by upstream kinases^26^; in addition, AMPK was recently reported to be synergistically activation by A-769662 and AMP, bypassing the need for threonine phosphorylation^27^. The UBA domain in AMPK (also referred to as AID) binds to the kinase domain and plays important roles in autoinhibition and allosteric activation of AMPK holoenzyme^28^,^29^. The KA1 domain of MARKs has been identified as membrane association module and may also regulate their kinase activities^30^,^31^,^32^,^33^. The C-terminal non-catalytic region of SAD kinases includes two short conserved regions, SCR1 and SCR2, which respectively contain the characteristic UBA and KA1 domains^1^,^2^,^7^,^31^. Yet, whether and how UBA and KA1 regulate SAD activity remains elusive.

In this study, structural and biochemical analyses of mouse SAD-A revealed a synergistic model for the autoinhibition of SAD activity by two separable elements, a newly identified autoinhibitory sequence (AIS) and the UBA domain. The assembly of a fully autoinhibited SAD kinase depends on the distinct intramolecular interactions among the kinase domain, the UBA domain and the AIS sequence, which provides the basis for developing specific inhibitors of SAD kinases, particularly targeting the unique pocket at kinase domain (KD)-UBA junction. We further applied the genetic approaches in *C. elegans* neurons and demonstrated that the cooperative autoinhibition by AIS and UBA in *C. elegans* SAD-1 is essential for presynaptic vesicle clustering. In addition, the AIS-KA1 fragment, but not the KA1 domain alone, can bind acidic phospholipids, which may localize SAD kinases to the membrane. We thus propose a model for the synergistic autoinhibition and membrane-binding triggered activation of SAD kinases.

## Results

### Two fragments of SAD-A autoregulate its kinase activity

**Figure 1:**
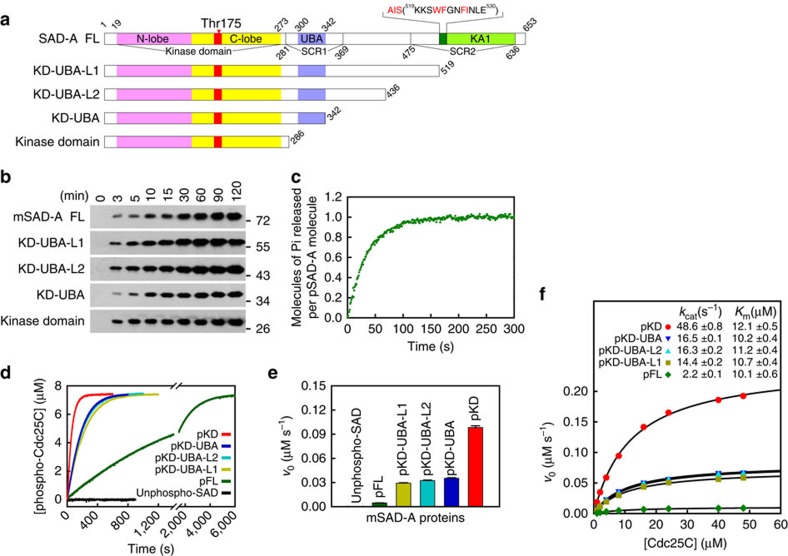
Two elements within the non-catalytic region autoinhibit SAD-A activity. (**a**) Schematic diagram of mouse SAD-A. The structural elements are coloured as follows: the kinase domain (N-lobe, pink; C-lobe, yellow; activation segment, red), UBA (slate blue), AIS (dark green) and KA1 (green). The sequence of AIS is provided, with the key residues highlighted in red. (**b**) Phosphorylation of SAD-A Thr175 by LKB1 analyzed using an anti-AMPK-pT172 antibody. (**c**) Representative time course of PP2Cα-catalyzed dephosphorylation of activated SAD. The reaction mixture contains 1 μM SAD-A full-length protein and 250 nM PP2Cα. (**d**) Time courses of SAD-catalyzed phosphorylation of Cdc25C. Reactions were initiated by adding 5 nM indicated SAD-A protein to the reaction mixture containing 7.5 μM Cdc25C peptide. (**e**) Comparison of the initial rates of 5 nM various SAD-A fragments towards 7.5 μM Cdc25C peptide (mean±s.e.m., *n*=3). (**f**) Plots of initial rate of SAD-catalyzed Cdc25C phosphorylation versus the Cdc25C concentration. The solid lines represent the best fitting results to the Michaelis–Menten equation with *k*_cat_ and *K*_m_ values listed at the top.

To dissect the precise function of the non-catalytic region of SAD kinases, we examined the effects of step-wise C-terminal truncations of mouse SAD-A on enzyme activity (Fig. 1a and Supplementary Fig. 1). Bacterially expressed SAD-A proteins were unphosphorylated, and the conserved Thr175 in the kinase activation loop could be readily phosphorylated by LKB1 (Fig. 1b). To quantitatively determine the phosphorylation stoichiometry of the SAD-A kinase domain, we employed a continuous spectrophotometric assay to monitor the dephosphorylation of SAD-A by PP2Cα. The reaction was near 100% complete in 2 min, and the phosphorylation stoichiometry was determined to be close to 1 mol of phosphate per mol of full-length SAD-A (Fig. 1c). Thus, LKB1 only phosphorylates the conserved Thr175 in the activation segment *in vitro*.

The unphosphorylated SAD-A proteins had no detectable enzymatic activity, while the LKB1-activated (Thr175 phosphorylated) SAD-A proteins can phosphorylate Ser216 of Cdc25C (ref. 12; Fig. 1d). Notably, the full-length SAD-A displayed a relatively low activity for Cdc25C phosphorylation (Fig. 1e). Removal of the sequence C terminal to the UBA domain by truncation at residues 519, 436 or 342 increased the initial velocity approximately sixfold, while further truncation to residue 286 resulted in an additional threefold increase. Thus, all C-terminal truncation mutants were more active than the full-length protein, and the kinase domain alone displayed the highest activity, ∼18-fold higher than that of the full-length SAD-A.

We then determined the steady-state kinetic parameters of various SAD-A mutants, each showing hyperbolic concentration dependence on the Cdc25C peptide (Fig. 1f). Consistent to the increased initial velocities, the C-terminal truncations resulted in striking increase in the *k*_cat_ values when compared with the full-length enzyme. However, little change was observed for the *K*_m(Cdc25C)_ values, indicating that the C-terminal regulatory region barely affects the binding of Cdc25C peptide to the substrate recognition site of SAD-A kinase domain. The *k*_cat_/*K*_m(Cdc25C)_ values for these SAD-A proteins were determined to be 0.22∼4.03 μM^−1^ s^−1^ and represent the largest values measured for protein kinases, indicating that Cdc25C is an efficient substrate of SAD-A. These kinetic data provide clear evidence for the presence of two autoinhibitory elements within the non-catalytic region of SAD-A, the UBA region (residues 286–342) and the C-terminal fragment (519–653), both of which regulate the rate of phosphoryl transfer.

### SAD-UBA binds to kinase domain in a distinct mode

**Figure 2:**
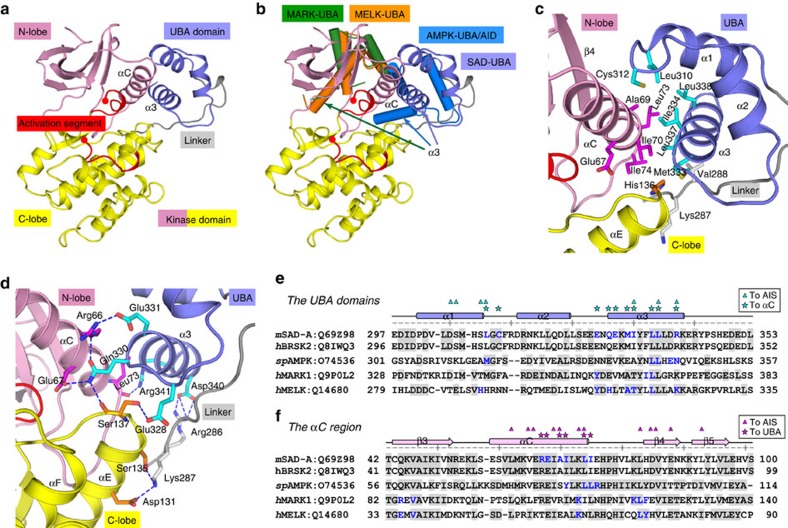
Distinct binding modes between SAD-A kinase and UBA domains. (**a**) Overall structure of SAD-A KD-UBA. The color scheme follows that in Fig. 1a. (**b**) Comparison of the KD-UBA structures from SAD-A, AMPK (PDB code: 3H4J), MARK1 (2HAK) and MELK (4IXP) upon superposition of kinase C-lobes. For clarity, only the UBA domains from AMPK, MARK1 and MELK are displayed. (**c**,**d**) Close-up views of the KD-UBA interface. The interacting residues from UBA are highlighted as cyan sticks, and those from the kinase N- and C-lobes are shown as magenta and orange sticks, respectively. Blue dashed lines represent the polar interactions. (**e**,**f**) Structure-based sequence alignments of the UBA domains and the αC regions from SAD-A, BRSK2, AMPK, MARK1 and MELK. Residues involved in respective KD–UBA interactions are highlighted in blue. Residues at the KD-UBA interface of SAD-A are also indicated by asterisks, and the AIS-interacting residues are indicated by triangles. The code following each protein name is the corresponding UniProt ID. m, mouse; h, human; sp, *Schizosaccharomyces pombe.*

To understand the autoinhibitory mechanisms, we first determined the crystal structure of mouse SAD-A fragment containing both kinase and UBA domains (Fig. 2a and Table 1). This KD-UBA structure comprises a head-to-tail dimer per asymmetric unit, which is probably a crystallization artifact owing to its monomeric state in solution (Supplementary Fig. 2a,b). The two molecules within an asymmetric unit adopt almost identical conformation, and the structural analyses hereafter were on the basis of monomer A with lower average temperature factors (Supplementary Fig. 2c,d). The kinase domain of SAD-A folds into a canonical bilobed structure in which the N-lobe consists of a β-sheet and the prominent αC helix and the C-lobe is composed mainly of α-helices. A flexible linker connects the catalytic domain to the UBA domain of three-helix bundle.

The AMPK kinase family is characterized by possessing the non-canonical UBA domain, and the UBA domains are structurally conserved in spite of the low sequence identity (Supplementary Fig. 2e,f). In all KD-UBA structures, the UBA domains bind to their respective kinase domains on the opposite face of the catalytic cleft^28^,^34^,^35^. However, the relative KD-UBA orientations are different (Fig. 2b). The UBA/AID domain of AMPK binds to both N- and C-lobes of the kinase domain, while those of MARKs and MELK interact exclusively with the N-lobes. Distinctly, the SAD-A kinase adopts a third binding mode, mainly with the N-lobe of the kinase domain and weakly with the C-lobe.

The KD–UBA interaction in SAD-A primarily involves two helices, α3 from the UBA domain and αC from the kinase domain, which buries ∼1,500 Å^2^ exposed surface area. Six conserved residues from the UBA domain pack against a hydrophobic patch at the C terminus of the prominent αC helix (Fig. 2c). The imidazole ring of His136 from the kinase αE helix also nestles into a hydrophobic pocket lined by residues from kinase αC, UBA α3 and the linker. In addition, several polar or charged side chains from the UBA domain and the linker form hydrogen bonds with residues from both kinase N- and C-lobes (Fig. 2d). For instance, Gln330 on UBA α3 tethers the two lobes by interacting with Glu67, Arg66 from helix αC and Ser137 C terminal to helix αE; in turn, Arg66 and Ser137 interact with two glutamates from UBA α3. Residues at SAD-A KD-UBA interface are conserved among SAD kinases; however, due to the different KD-UBA binding modes the interacting residues vary from one AMPK family member to another (Fig. 2e,f and Supplementary Fig. 1). In particular, Glu331 on helix α3 of the SAD-A UBA domain is charged and involved in KD–UBA interactions, while the corresponding residues in all other AMPK-RKs are hydrophobic and essential for integral of the UBA domains (Fig. 2d and Supplementary Fig. 2f). Therefore, the UBA α3 helix similarly predominates the KD–UBA interaction in AMPK family members, yet the binding modes and interacting residues are distinct.

### Binding of UBA orients helix αC in an inactive conformation

**Figure 3:**
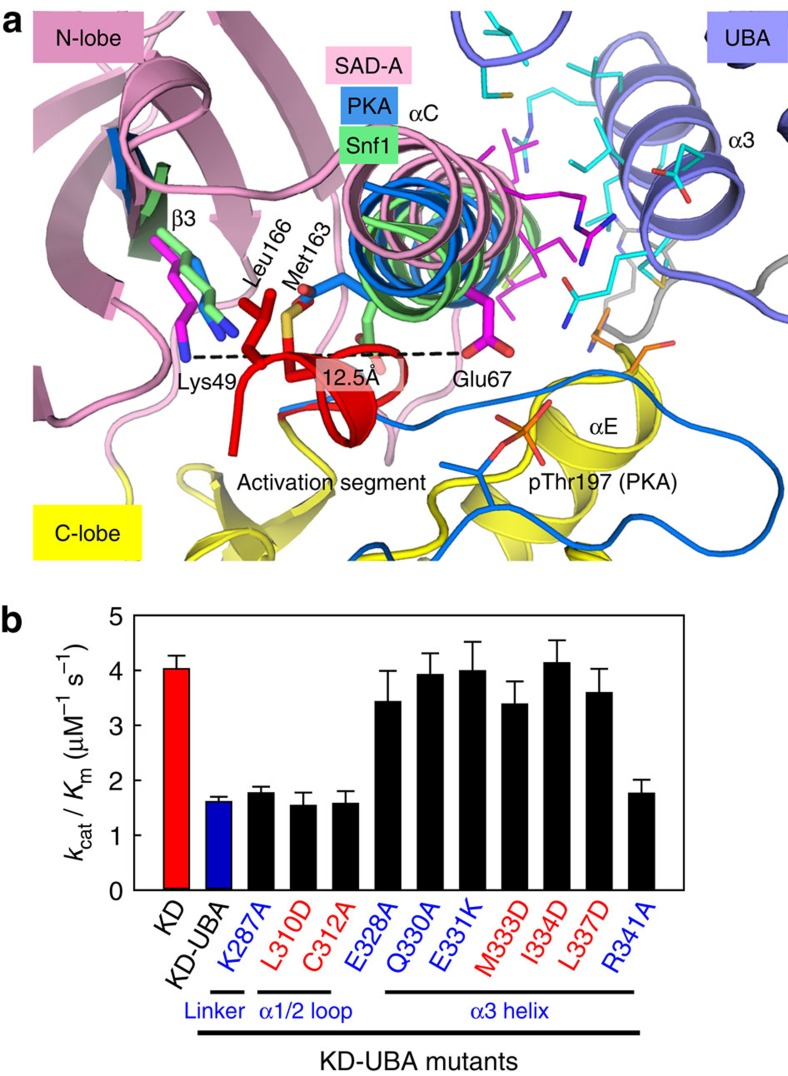
Binding of UBA immobilizes the αC-out kinase conformation. (**a**) Comparison of the autoinhibited SAD-A with the active conformations of PKA (PDB code: 1ATP) and Snf1 (2FH9). For clarity, only strands β3, helices αC and activation segments of PKA (marine blue) and Snf1 (green) are displayed. The conserved Lys and Glu side chains and two hydrophobic residues in the activation segment of SAD-A are highlighted as sticks. (**b**) Effects of the UBA mutations on the catalytic efficiencies of KD-UBA. The *k*_cat_/*K*_m_ values are determined as that in Supplementary Table 1.

The UBA domains from different AMPK family members regulate the kinase activities through distinct mechanisms. The AMPK-AID directly inhibits the kinase domain by constraining the mobility of helix αC (ref. 28). By contrast, the UBA domains of MARKs and MELK indirectly regulate the catalytic activities by enhancing LKB1-activation or promoting proper folding^35^,^36^. Consistent with the structural observation that SAD-A largely resembles AMPK, we found the SAD-UBA directly and specifically inhibits the activity of SAD-A kinase domain (Supplementary Fig. 3a). Structural comparison reveals that the KD–UBA interaction in SAD-A brings helix αC into an orientation that is rotated outwards approximate 90° from that in the active kinases including AMPK ortholog Snf1 and the prototype PKA (Fig. 3a). The N-terminal portion of the partially disordered activation segment folds back into the active site, strictly preventing the formation of salt bridge(s) between Glu67 on helix αC and Lys49 on strand β3. Two conserved hydrophobic side chains of Met163 and Leu166 form multiple contacts with residues from the β-sheet and αC helix, further stabilizing the displaced conformation (Supplementary Fig. 3b). Therefore, the distinct UBA binding mode and the inserted activation segment lead to a typically inactive (αC-out) kinase conformation, where the important Glu67 is 12.5 Å away from Lys49.

To assess the UBA inhibition, we generated a series of point mutations on the KD-UBA fragment of SAD-A and evaluated their effect on kinase activity (Fig. 3b and Supplementary Table 1). When the interacting residues on or near the UBA α3 helix were individually substituted, the catalytic efficiencies of all mutants, but R341A, were increased two to threefold, comparable to that of the kinase domain alone; in contrast, substitution of the amino acids on the linker and α1-α2 loop had little, if any, effect on basal activity. Similar to the truncation results shown in Fig. 1f, the effective UBA mutations led to increase in the *k*_cat_ values but little change in the *K*_m(Cdc25C)_ values (Supplementary Table 1). These results corroborate that the UBA α3 helix plays a predominant role in displacing the orientation of helix αC and thereby inhibiting the catalytic activity, particularly the phosphoryl transfer step, of SAD kinase domain.

### The AIS binds to KD-UBA junction

**Figure 4:**
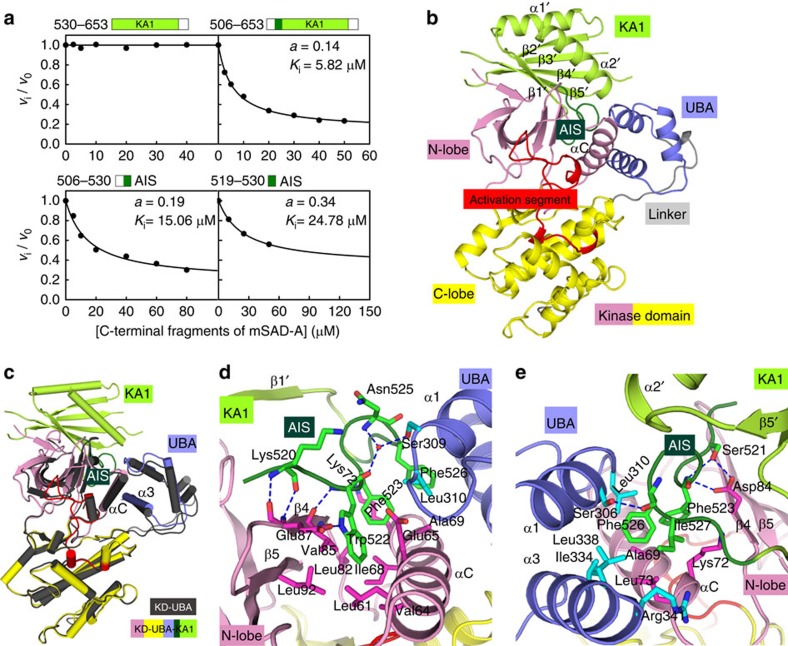
AIS binds at the KD-UBA junction and inhibits SAD-A activity. (**a**) *Trans*-inhibition of different C-terminal fragments on the activity of KD-UBA (10 nM). The continuous curves were the best-fit to the non-competitive model using equation *v*_i_/*v*_0_=(*K*_i_+*a*[*I*])/(*K*_i_+[*I*]), where *K*_i_ and *a* are the apparent inhibition constant and residual activity, respectively. (**b**) Overall structure of the KD-UBA and AIS-KA1 complex. The color scheme for the complex follows that in Fig. 1a. (**c**) Comparison of the KD-UBA conformations in complex with AIS-KA1 and in isolation. The KD-UBA alone is shown in dark grey. (**d,e**) Close-up views of AIS binding to the KD-UBA junction. The interacting residues in the AIS sequence are highlighted as green sticks, and those from the kinase and UBA domains are shown as magenta and cyan sticks, respectively.

In addition to the UBA domain, the C-terminal fragment (residues 519–653) of SAD-A can remarkably reduce the SAD activity (Fig. 1). The C-terminal regions of the yeast SAD kinase Hsl1 and certain AMPK family members were suggested to exert regulatory function as well^32^,^33^,^37^,^38^,^39^. This additional inhibitory fragment of SAD-A contains a KA1 domain and two flanking loops, and we carried out *trans*-inhibition assays to determine the precise inhibitory segment (Fig. 4a and Supplementary Fig. 4a). Unexpectedly, neither the KA1 domain alone (530–640) nor the fragment including the C-terminal loop (530–653) exhibited inhibitory effect on the catalytic activity of KD-UBA. By contrast, the SAD-A fragment or the chimeric protein containing the sequence N terminal to KA1 (506–530) effectively *trans*-inhibited the kinase activity. These two proteins incompletely inhibited the KD-UBA activity, plateauing at ∼15%, which is consistent with the approximately sixfold inhibition in the truncation experiments (Fig. 1). Similarly, the shorter fragment containing residues 519–653, albeit with low solubility, suppressed the KD-UBA activity with comparable efficiency (Supplementary Fig. 4a). To confirm the significance of the segment N terminal to the KA1 domain, we synthesized two peptide of residues 506–530 and 519–530, and both peptides *trans*-inhibited the KD-UBA activity (Fig. 4a). These data clearly demonstrated that the sequence ^519^KKSWFGNFINLE^530^ exerts an inhibitory effect on SAD activity. This segment is hereafter referred to as the AIS.

To unravel the regulatory mechanism of AIS, all the AIS-containing fragments in complex with KD-UBA were subjected to crystallization trials, and the complex structure containing the AIS-KA1 fragment of residues 519–653 was determined to 2.5 Å resolution (Table 1). The UBA similarly binds to the kinase domain from backside, and the AIS-KA1 fragment sits on the top of KD-UBA (Fig. 4b). Comparison of two KD-UBA conformations, in isolation and in complex with AIS-KA1, revealed that the AIS-KA1 binding does not induce a large rearrangement of the kinase domain, and that the major inactivating effect is likely due to the influence of AIS on the αC conformation (Fig. 4c).

Consistent with the *trans*-inhibition results, the non-inhibitory KA1 domain makes few contacts with KD-UBA (Supplementary Fig. 4b). Contrarily, the AIS sequence, adopting an extended conformation, makes many interactions with both the kinase and UBA domains (Fig. 4d,e and Supplementary Fig. 4c). Two consecutive aromatic residues from the AIS, Trp522 and Phe523, penetrate into a hydrophobic crevice between the β-sheet and αC helix of kinase domain. The following Phe526 and Ile527 stick into an adjacent hydrophobic pocket formed by residues from three helices, the kinase αC and the UBA α1, α3. The conformation of AIS is further stabilized by multiple hydrogen bonds, directly or water mediated; in particular, Trp522 and Ser521 from the AIS interact with two acidic residues Glu87 and Asp84 on the kinase β4 strand. The extensive interactions ensure the stable complex between KD-UBA and AIS-KA1 and reinforce the inactive conformation of the essential αC helix. More importantly, the AIS sequence pries away the N-lobe β-sheet from the αC helix, resulting in further separation of the pivotal Lys49 and Glu67 (Supplementary Fig. 4d). These structural observations and the incomplete *trans*-inhibition provide evidence for a non-competitive manner of AIS inhibition.

### UBA and AIS synergistically autoinhibit SAD activity

**Figure 5:**
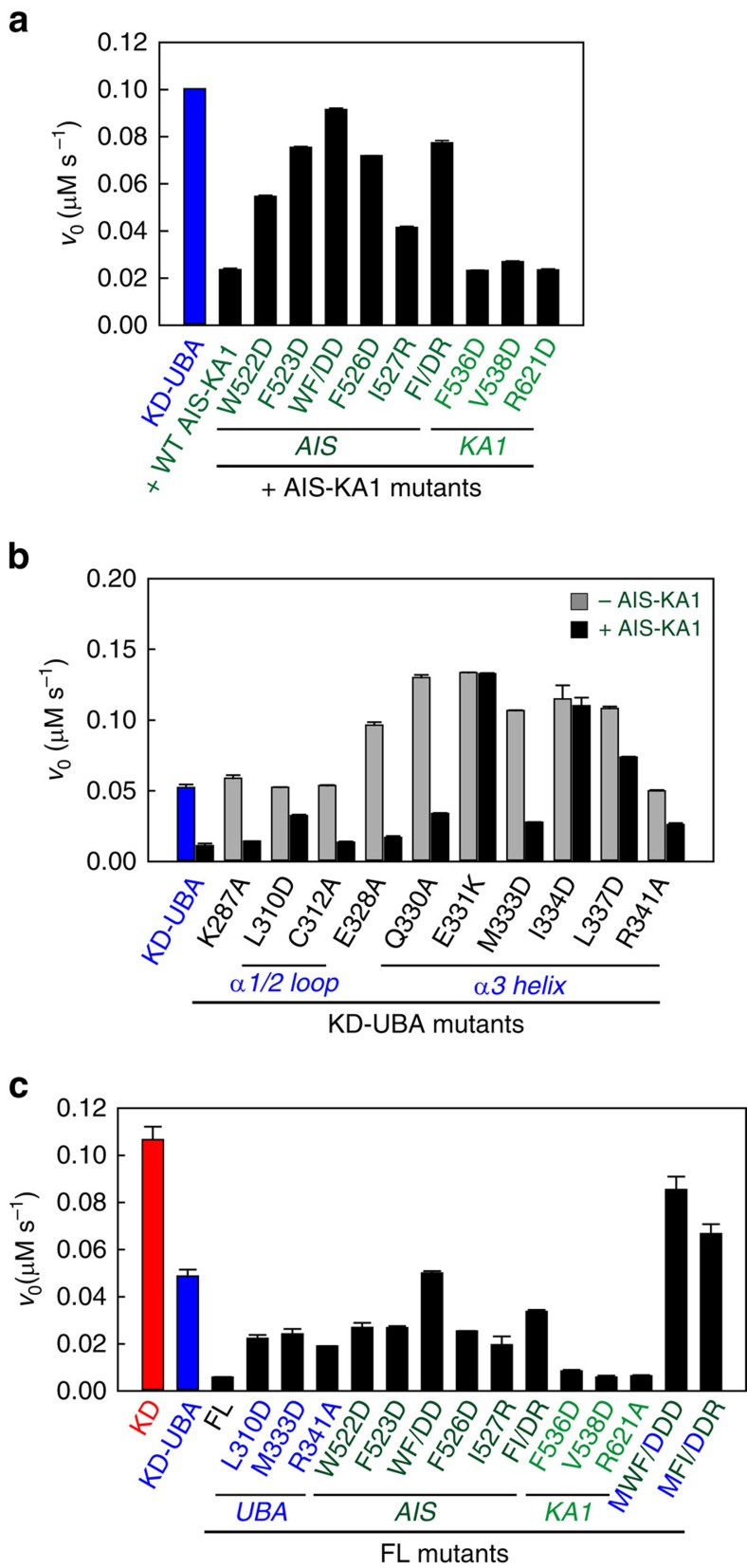
AIS and UBA synergistically regulates SAD activity. (**a**) Effects of the AIS and KA1 mutations on the *trans*-inhibition of AIS-KA1 on the KD-UBA activity. The assays were performed with 10 nM wild-type KD-UBA and 50 μM AIS-KA1 mutants. (**b**) Comparison of the catalytic activities of various KD-UBA mutants in the absence or presence of AIS-KA1. The assays were performed with 5 nM KD-UBA mutants and 50 μM wild-type AIS-KA1. (**c**) Activities of full-length SAD-A bearing various UBA and/or AIS mutations. Reactions were initiated by adding 5 nM indicated SAD-A proteins. (mean±s.e.m., *n*=3)

To confirm the AIS autoinhibition, we first generated various AIS and KA1 mutations on the AIS-KA1 fragment and examined their effects on the *trans*-inhibition on wild-type KD-UBA (Fig. 5a). Individually mutating four central hydrophobic residues of AIS to charged side chains largely abolished the AIS inhibition on the KD-UBA activity; however, substitutions of three interface residues on the KA1 domain had no effects. These data corroborated that it is the AIS, but not the KA1 domain, that exhibits the inhibitory effect.

Unexpectedly, the AIS-KA1 fragment did not inhibit the activity of kinase domain alone, indicating that the AIS autoinhibition on SAD activity requires the UBA domain (Supplementary Fig. 5). To examine the importance of UBA, we then carried out *trans*-inhibition assay with various KD-UBA proteins bearing UBA mutations (Fig. 5b). Indeed, mutations of several essential KD-UBA interface residues (E331K, I334D and L337D) completely or greatly abolished the AIS inhibition. However, the AIS retained evident inhibition on activities of some KD-UBA mutants (E328A, Q330A and M333D) in spite of their dramatic effects on UBA autoinhibition. One potential explanation is that the AIS makes interactions with both the kinase and UBA domains, which partially compensates the effect of these mutations. Notably, substitution of Leu310 and Arg341 also exhibited marked effects on the AIS *trans*-inhibition, notwithstanding their negligible effects on KD-UBA interaction. This can be attributed to the direct interactions between these two UBA residues and three central AIS residues (Fig. 4d,e). These data indicated that the proper association between the kinase and UBA domains of SAD-A is essential for the AIS inhibition. Thus, the UBA domain has dual functions in the autoinhibtion of SAD activity, directly inhibiting the activity of kinase domain and serving as an indispensable part of the AIS function.

To further assess the synergistic inhibition of AIS and UBA, we generated a series of point mutations on full-length SAD-A (Fig. 5c). Substitutions of most of the key UBA residues dramatically reduced the solubility of the full-length proteins, suggesting that SAD-UBA might contribute to the folding of nascent polypeptide, reminiscent of MELK (ref. 35). The soluble UBA mutants displayed 3–4-fold increase of catalytic activity compared with the wild-type enzyme, yet they were less active than the KD-UBA fragment owing to the intramolecular inhibition by AIS. When the four essential AIS residues were substituted, the catalytic efficiencies of the single or double mutants were evidently enhanced, as that observed in the *trans*-assay shown in Fig. 5a. Particularly, the double mutation of Trp522 and Phe523 (WF/DD) exhibited the same activity as the KD-UBA fragment, completely abolishing the AIS inhibition. We also generated triple mutations to release both the UBA and AIS inhibitions, and these mutants indeed yielded dramatic increase in the SAD activity, comparable to that of the kinase domain alone. By contrast, the KA1 mutations had no effect. Taken together, the structural and biochemical data demonstrated that the AIS and UBA synergistically inhibit the SAD activity by orienting helix αC into an inactive conformation.

### Synergistic autoinhibition is essential for SAD function

**Figure 6:**
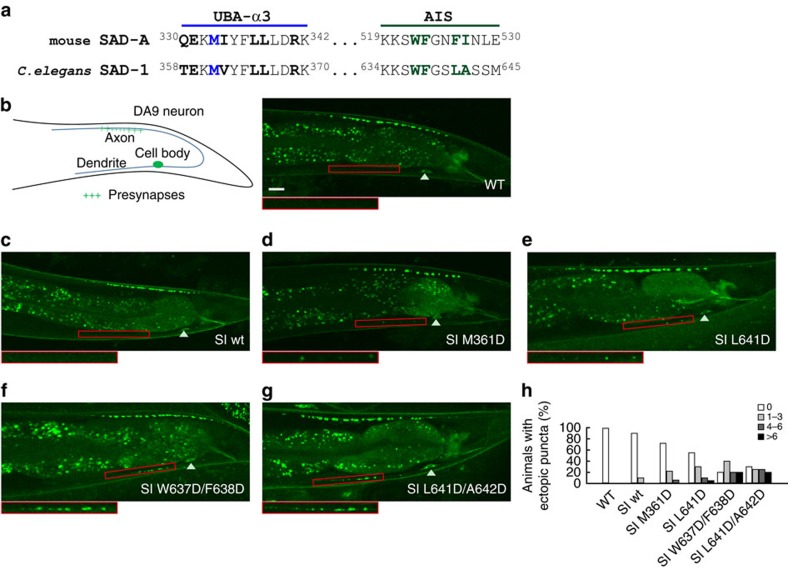
*C. elegans* SAD-1 mutations mislocate presynaptic vesicle clusters to dendrite of DA9 neuron. (**a**) Sequence alignment of UBA-α3 and AIS from mouse SAD-A and *C. elegans* SAD-1. Residues involved in intramolecular interactions are shown in bold, and those mutated in following assays are highlighted in blue or green. (**b**) Localization of synaptic vesicle-associated GFP::RAB-3 in wild-type animals. (**c–g**) Distribution of GFP::RAB-3 in animals expressing single-copy insertion of wild-type and mutant *sad-1*. Scale bar, 10 μm. (**h**) Quantification of ectopic dendritic puncta in wild-type and mutant animals. The columns represent the percentage of animals with 0, 1–3, 4–6, or >6 extra presynaptic puncta in the dendrite.

Considering the highly conserved sequence and the identical domain organization, we proposed that the synergistic autoinhibition observed in mouse SAD-A would most likely be conserved in *C. elegans* SAD-1 (Fig. 6a and Supplementary Fig. 1). Indeed, the AIS sequence from SAD-1 can efficiently inhibit the KD-UBA activity *in vitro* (Supplementary Fig. 6a). SAD-1 was known to regulate neuronal polarity and synaptic organization in *C. elegans*, and the kinase activity was required for its functions^1^,^40^. We then analyzed presynaptic vesicle clustering in DA9 motor neuron using GFP::RAB-3 as marker. In wild-type worm, the synaptic vesicles were restricted to the axon and no puncta were detected at the dendritic region^41^ (Fig. 6b). Overexpression of *sad-1* led to increased SAD-1 activity and thereby stimulated the ectopic accumulation of synaptic vesicles in the DA9 dendrite, similar to that observed in ASI neuron^1^ (Supplementary Fig. 6b). To examine the importance of synergistic autoinhibition, we mutated the conserved interacting residues on UBA α3 helix or in the AIS sequence, and introduced a single-copy insertion of *sad-1* into *C. elegans*. Animals bearing the single-copy insertion of wild-type *sad-1* (SI wt) displayed similar phenotype as wildtype (Fig. 6c). Imaging analysis of *sad-1* point mutations (SI M361D and SI L641D) revealed ectopic vesicle clusters in the dendrite (Fig. 6d,e), while the double mutants (SI W637D/F638D and SI L641D/A642D) caused more severe polarity defects (Fig. 6f,g). Barely 30% of the single mutant animals display the ectopic phenotype with less than three ectopic dendritic puncta; by contrast, ∼50% of animals expressing the double mutants have more than four ectopic puncta (Fig. 6h). In addition, we determined whether these mutants could rescue the DA9's phenotype in the *sad-1*(*ky289*) protein-null animals (Supplementary Fig. 6c). As expected, the wild-type *sad-1* restored both synaptic organization at the distal end of DA9's axon and neuronal polarity in the dendrite region^1^,^42^,^43^,^44^. The UBA and AIS mutants, by contrast, rescued synaptic organization defect but not neuronal polarity defect. The ectopic accumulations of synaptic vesicles in the mutant rescue experiments were likely due to the increased activities of these *sad-1* mutants. Together, the *in vitro* and *in vivo* data demonstrated that the SAD activity was cooperatively regulated by AIS and UBA, and this synergistic autoinhibition is of great importance for axonal–dendritic polarity.

To date, there are 5,911 reported single-nucleotide polymorphisms (SNPs) for human BRSK2/SAD-A (http://www.ncbi.nlm.nih.gov/projects/SNP/snp_ref.cgi?geneId=9024) and 2,262 for BRSK1/SAD-B (http://www.ncbi.nlm.nih.gov/projects/SNP/snp_ref.cgi?geneId=84446). In particular, seven UBA and one AIS residues of *h*BRSK2 and seven UBA and five AIS residues of *h*BRSK1 are altered (Supplementary Fig. 6d). Most of the SNPs would have little effect on the UBA and AIS autoinhibition, including the conserved substitution of the important AIS residues, human BRSK2 F525L and BRSK1 I600V (corresponding to mouse SAD-A Phe526 and Ile527). However, the human BRSK1 S322G and BRSK2 S594C mutations might interfere with the UBA/AIS functions, since the side chains of corresponding *m*SAD-A residues Ser306 and Ser521 are involved in the UBA–AIS and AIS–KD interactions, respectively (Fig. 4e). In addition, the human BRSK1 V319D, F329C and BRSK2 M306V variations, respectively, corresponding to the substitutions of *m*SAD-A Val303, Phe313 and Met307, would disrupt the intra-UBA hydrophobic interactions and thus destabilize the UBA structure. Notably, the V319I mutation of human BRSK1 was reported in the large cell carcinoma of the lung^45^. Therefore, some SNPs might affect the UBA and AIS function, and more studies are needed to explore their physiological significances.

### Both AIS and KA1 are indispensable for phospholipid binding

**Figure 7:**
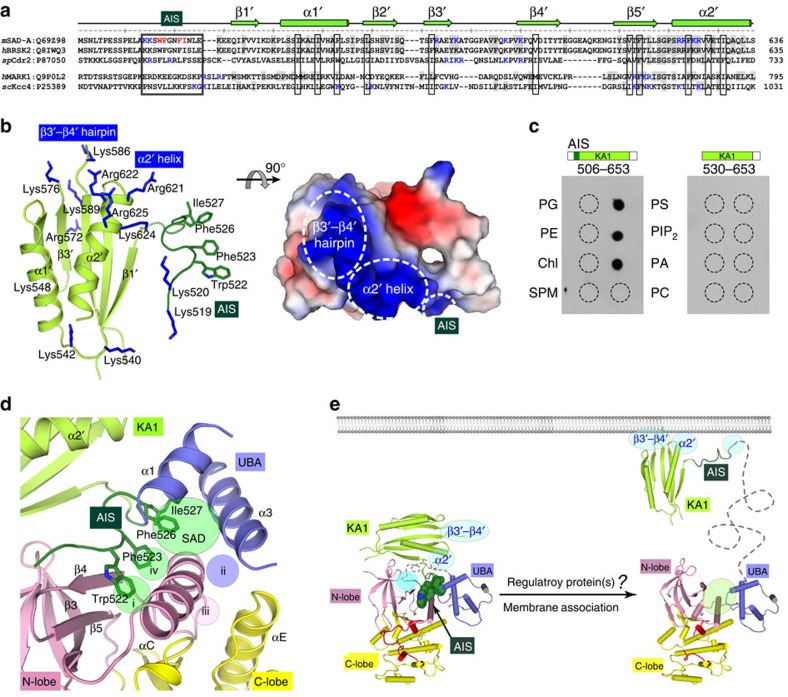
Model for the autoinhibition and activation of SAD-A. (**a**) Sequence alignment for the AIS-KA1 regions of SAD and MARK kinases. The positively charged residues required for phospholipid binding are highlighted in blue. h, human; m, mouse; sp, *Schizosaccharomyces pombe;* sc, *Saccharomyces cerevisiae*. (**b**) Three basic clusters in the AIS-KA1 fragment. The basic residues are shown as blue sticks, and the surface representation is coloured according to electrostatic potential (positive, blue; negative, red). (**c**) Lipid-binding assays for two representative C-terminal fragments. PG, phosphatidylglycerol; PE, phosphatidylethanolamine; Chl, cholesterol; SPM, sphingomyelin; PS, phosphatidylserine; PIP_2_, phosphatidylinositol-4,5-bisphosphate; PA, phosphatidic acid; PC, phosphatidylcholine. (**d**) Conserved (i–iv) and specific (SAD) hydrophobic pockets surrounding helix αC. Three AIS-binding pockets are indicated by green circles, and that for UBA binding is coloured in blue. For clarity, only helices α1 and α3 of UBA are displayed. (**e**) Regulation model for SAD-A. The extensive intramolecular interactions keep SAD-A in an autoinhibited conformation, where the kinase activity is synergistically inhibited by UBA and AIS. Membrane association of the AIS-KA1 fragment, probably triggered by regulatory protein(s), might release the AIS (and UBA) inhibition.

The KA1 domain of SAD-A folds into two helices and a five-stranded β-sheet, which, despite the low sequence homology, is structurally conserved to the KA1 domain of MARKs and the C-terminal domain of AMPK α-subunits^46^,^47^ (Fig. 7a,b and Supplementary Fig. 7a,b). Since the KA1 domain of MARKs can mediate membrane association, we performed *in vitro* protein-lipid overlay assays with various mouse SAD-A C-terminal fragments (Fig. 7c). The C-terminal fragments of SAD-A, including both the AIS sequence and the KA1 domain, were able to bind to acidic phospholipids, such as phosphatidylserine, phosphatidylinositol-4,5-bisphosphate and phosphatidic acid. Unexpectedly, the KA1 domain alone failed to bind to phospholipids, which indicated that the AIS sequence is required for lipid binding of SAD-A.

Certain surface-exposed basic residues from the KA1 domains are required for membrane association of MARK and Cdr2 kinases^31^,^48^; however, the distribution of positively charged patches on SAD-A KA1 domain is different (Fig. 7b and Supplementary Fig. 7b). There are two spatially adjacent clusters of basic residues at one end of the KA1 domain, one in the β3′-β4′ hairpin and the other at the N terminus of helix α2′. In addition, the AIS sequence contains two consecutive basic residues (Fig. 7b). We then validated the importance of these basic clusters for lipid binding (Supplementary Fig. 7c). Replacement of the positively charged side chains in each clusters by uncharged Ser led to complete lipid detachment of the SAD-A AIS-KA1 fragment; by contrast, substitution of three discrete basic residues on the other end of SAD-KA1 had no effect on lipid interaction. These results suggest that the phospholipid binding and likely membrane association of the SAD kinases require three basic clusters within the AIS-KA1 fragment, and, more importantly, the AIS sequence is the key regulatory element of SAD autoinhibition and lipid binding.

## Discussion

Protein kinases are important regulators of intracellular signal transduction, and the precise regulation of kinase activity is essential for normal cellular functions. Most kinases have multiple regulatory elements, among which the *cis*-acting inhibitory element or autoinhibitory sequence is the major mechanism for negative regulation of kinase activities^19^,^49^,^50^,^51^. In this study, we identified two autoregulatory elements within the non-catalytic region of SAD kinases, the conserved UBA domain and a unique AIS sequence. The SAD-UBA domain binds to kinase domain in a distinct mode from those observed in other AMPK family members, and partially inhibits the SAD activity (Figs 2 and 3). The newly identified AIS binds to the specific KD-UBA junction and significantly inhibits kinase activity in cooperation with UBA (Figs 4 and 5). This AIS sequence, N terminal to the KA1 domain, is highly conserved among SAD kinases ranging from yeast to human, but diverse in all other AMPK family members (Supplementary Figs 1 and 7a). Thus, we propose that the synergistic autoinhibition of AIS and UBA is unique to the SAD kinases. However, it is an open question whether the variant linkers between the UBA domain and the AIS sequence and the divergent C-terminal tails following the KA1 domain play certain role(s) in SAD regulation.

Several molecular mechanisms have been described for kinase regulation, such as restricting the orientation of helix αC, hindering the binding site for ATP or substrate and modulating the conformation of activation segment^51^,^52^,^53^,^54^. In SAD-A, AIS and UBA synergistically act on helix αC, immobilizing the kinase domain in an ‘αC-out' inactive conformation or slowing down the transition rate from inactive to active form. Noteworthy, this extensive interface involves three out of four spatially conserved pockets (i, ii and iv) on helix αC, presenting in different kinase branches of the kinome tree^55^ (Fig. 7d). The aromatic rings of two highly conserved AIS residues Trp522 and Phe523, respectively, penetrate into pockets i and iv, and several hydrophobic residues from SAD-UBA insert into pocket ii. More importantly, the other two essential residues of AIS, Phe526 and Ile527, nestle into a specific binding pocket between the kinase and UBA domains (Fig. 7d). This long, narrow AIS-binding site, including the conserved αC pockets i, iv and the specific KD-UBA interface pocket, is present only in SAD kinases. The UBA domains from AMPK, MARK and MELK would sterically clash with the bound AIS of SAD-A, especially residues Phe526 and Ile527 (Supplementary Fig. 7d). Consistently, the AIS sequence of SAD-A cannot inhibit the kinase activities of the KD-UBA fragments from AMPK and other AMPK-RKs (Supplementary Fig. 7e). This unique autoinhibition by AIS provides the basis for developing specific inhibitors of SAD kinases, particularly targeting the specific pocket at the junction of kinase and UBA domains.

Release of autoinhibition can occur by several mechanisms, including post-translational modification, interaction with regulatory molecules and lipid/membrane association^19^,^56^,^57^,^58^. The membrane association of MARK via its KA1 domain has been suggested to coincide with the increase of kinase activity^30^,^31^. SIK2 can co-localize with the p97/VCP ATPase to ER membrane and regulate ER-associated protein degradation^59^. Recently, evidence suggested that AMPK locates on the endosomal surface where it can be activated by LKB1 at lower AMP threshold concentrations on energy stress^60^. Thus, membrane localization might be a common mechanism for the regulation and function of AMPK family members. The SCR2 region of human BRSK1/SAD-B has been found associated with synaptic vesicles, and the C-terminal fragment of *Schizosaccharomyces pombe* SAD kinase Cdr2 was required for its anchoring to the cortex^7^,^48^. Since the AIS-KA1 fragment of SAD-A can interact with acidic phospholipids (Fig. 7c), we believe that AIS-KA1 might target SAD-A to particular membrane locations. However, all three basic clusters required for lipid binding are sterically buried or hindered in the autoinhibited SAD, which suggests that the membrane association of AIS-KA1 would drag the AIS away from the KD-UBA junction and thereby activate, at least partially, the SAD kinase (Fig. 7e). To verify this hypothesis, we then carried out the protein-lipid overlay assay with full-length SAD-A. Unexpectedly, the full-length protein did not interact with phospholipids (Supplementary Fig. 7f). Moreover, when the AIS-KA1 fragment was preincubated with KD-UBA, the complex of AIS-KA1 and KD-UBA did not bind phospholipids either. These data suggest that the presence of the KD-UBA fragment can block the lipid binding of AIS-KA1, or the AIS-KA1 fragment in the intact SAD kinase prefers to interact with KD-UBA, rather than phospholipids or membrane.

Then, how would SAD kinases be activated and located to membrane? The C-terminal domain of AMPK α-subunit mediates assembly of AMPK holoenzyme by interacting with the β- and γ-subunits (Supplementary Fig. 7b). The scaffold protein GAB1 and death-associated protein kinase can bind the non-catalytic region of MARKs and regulate their kinase activities^32^,^33^. Moreover, the synaptic protein neurabin can bind to the C-terminal non-catalytic region of *C. elegans* SAD-1 *in vivo* and *in vitro*, and this physical interaction plays a role in regulating neuronal polarity^42^. The cytoskeletal septin filaments had also been proved to directly associate with the C-terminal region of *Saccharomyces cerevisiae* SAD ortholog Hsl1, which localizes Hsl1 to the bud neck and relieves its autoinhibition^37^. We thus speculate that the certain regulatory proteins might bind to the non-catalytic region of SAD kinases, alter the intramolecular interactions between AIS, UBA and kinase domain, and lead to relief of the AIS (and UBA) inhibition. Then, three exposed basic clusters in the free AIS-KA1 fragment can cooperatively bind to acidic phospholipids, resulting in a membrane bound, activated SAD kinase (Fig. 7e). Indeed, the SAD-A W522D/F523D mutant alone was capable of binding to acidic phospholipids in the protein-lipid overlay assay, confirming that the AIS-KA1 fragment in the AIS-released/free full-length SAD-A plays a *bona fide* phospholipid binding role (Supplementary Fig. 7f). Based on the results presented in this study and knowledge of AMPK family, we propose this synergistic autoinhibition and lipid/membrane activation model for the SAD-A/BRSK2 kinases. We are keenly interested in the remaining questions, such as which regulatory protein(s) could release the synergistic autoinhibition by UBA and AIS and trigger membrane association of SAD kinases, and whether drugs could activated SAD-A independently of the Thr175 phosphorylation, similar to that reported for AMPK (ref. 27).

## Methods

### Protein preparation

The complementary DNAs of mouse SAD-A and *C. elegans* SAD-1 were kindly provided by Dr Joshua R. Sanes (Harvard University) and Dr Mei Zhen (University of Toronto), respectively. The full-length SAD-A and various fragments were amplified by standard PCR and cloned into pGEX-6P-1 and/or pET21b vectors with N-terminal GST-tag or C-terminal His_6_-tag. The short segments containing the AIS sequences of mouse SAD-A (residues 506–530) and *C. elegans* SAD-1 (621–645) were fused to a C-terminal thioredoxin-tag to generate high-quality peptides. All site-specific mutations were generated by overlap PCR procedure and verified by DNA sequencing. The plasmids of human LKB1, STRADα and MO25α were kindly provided by Dr Gail Fraser and Dr Maria Deak (University of Dundee), respectively. The active LKB1-STRAD-MO25 complex was similarly expressed in Sf9 cells and purified^61^. The human pET28a-PP2Cα expression plasmid was a generous gift from Dr Mark Solomon (Yale University).

All proteins, overexpressed in *Escherichia coli* BL21 (DE3) cells at 18 °C, were purified over Ni-NTA (Qiagen) or GS4B (GE Healthcare) columns, followed by ion exchange and gel filtration chromatography (Source-15Q/15S and Superdex-200/75, GE Healthcare). The purified proteins in a buffer containing 10 mM HEPES, pH 7.4, 150 mM NaCl and 2 mM DTT were stored at −80 °C and subjected to crystallization trials. Protein stocks used for enzymatic assay were supplemented with glycerol at a final concentration of 20% (v/v).

### Phosphorylation of SAD-A by LKB1

The SAD-A proteins (5 μM) were incubated with the LKB1 complex (10 nM) in a phosphorylation buffer containing 50 mM MOPS, pH 7.0, 2 mM DTT, 100 mM NaCl, 10 mM MgCl_2_ and 1 mM ATP at 25 °C. The reactions are initiated by adding LKB1. At indicated time intervals, aliquots were removed from the reaction mixture and phosphorylation was terminated by adding EDTA to a final concentration of 50 mM. The samples were then subjected to western blot analysis to examine the phosphorylation state of the conserved Thr175 in the kinase activation loop of SAD-A using an antibody against the phospho-Thr172 of AMPK (1:5,000; Cell Signaling Technologies, #2535). The uncropped blots were shown in Supplementary Fig. 8.

The maximum phosphorylation was observed after 60 min. To generate fully phosphorylated proteins, various SAD-A fragments or mutants (5 μM) were incubated with 50 nM LKB1 and 1 mM ATP for at least 60 min. The samples were then subjected to a coupled kinase assay for SAD-A by using a Cdc25C peptide as substrate (see below).

### Dephosphorylation of SAD-A by PP2Cα

Dephosphorylation of the LKB1-activated SAD-A proteins by PP2Cα was measured using a continuous spectrophotometric assay^62^. The assay was performed at 25 °C in a coupled enzyme system containing 50 mM MOPS, pH 7.0, 100 mM NaCl, 0.1 mM EDTA, 10 mM MgCl_2_, 100 μM 7-methyl-6-thioguanosine (MESG, Berry & Associates), and 0.1 mg ml^−1^ purine nucleoside phosphorylase (Sigma). This coupled system uses purine nucleoside phosphorylase and its chromogenic substrate MESG to monitor the production of inorganic phosphate. The reactions were initiated by adding PP2Cα, and the continuous absorbance changes at 360 nm were recorded with a PerkinElmer LAMBDA 45 spectrophotometer equipped with a magnetic stirrer in the cuvette holder. The concentration of MESG was determined at 331 nm using a molar extinction coefficient of 32,000 M^−1^ cm^−1^, and quantification of phosphate release was measured at 360 nm using the extinction coefficient of 11,200 M^−1^ cm^−1^ (ref. 63).

### Kinetic analysis of SAD kinases

The kinase activity of SAD-A was determined using the synthetic Cdc25C peptide (^201^DQEAKVSRSGLYRSP***S***MPENLNRPRLKQVE^230^, SciLight Biotechnology) as substrate. The standard assay was performed with 5 nM SAD-A protein (full-length, fragments or mutants) and indicated amounts of the Cdc25C peptide at 25 °C, in 1.8-ml reaction mixture containing 50 mM MOPS, pH 7.0, 100 mM NaCl, 0.1 mM EDTA, 10 mM MgCl_2_, 1 mM ATP, 200 μM NADH, 1 mM phosphoenolpyruvate, 20 U ml^−1^ lactate dehydrogenase, and 15 U ml^−1^ pyruvate kinase. This spectrophotometric kinase assay couples the production of ADP with the oxidation of NADH by pyruvate kinase and lactate dehydrogenase^28^. Progress of the reaction was monitored continuously by following the decrease of NADH at 340 nm on the PerkinElmer Lambda 45 spectrophotometer, and the initial rates were determined from the linear slopes of the progress curves. The kinetic parameters (*k*_cat_ and *K*_m_) were obtained by fitting the experimental data to the Michaelis–Menten equation using a nonlinear regression analysis programme. The concentrations of ADP generated in the SAD-catalyzed reaction were calculated with an extinction coefficient for NADH of 6,220 M^−1^ cm^−1^ at 340 nm, and concentration of the Cdc25C peptide was determined by turnover with the activated SAD-A under condition of limiting Cdc25C peptide.

The *trans*-inhibition studies were performed at 25 °C in the 1.8-ml kinase reaction mixture with indicated inhibitory fragments containing the UBA domain or the AIS sequence. The AIS peptide (^519^KKSWFGNFINLE^530^) and the AIS-containing peptide (^506^MSNLTPESSPELAKKSWFGNFINLE^530^) were synthesized using standard protocol, purified by reverse-phase preparative HPLC and characterized by matrix-assisted laser desorption/ionization–time of flight mass spectrometry by Scilight Biotechnology Ltd. (Beijing, China). The enzyme, the kinase domain alone or the KD-UBA fragment of SAD-A, was preincubated with indicated amount of inhibitory SAD fragment in the reaction mixture for 15 min. The reaction was initialized by adding the substrate Cdc25C peptide (20 μM), and the initial rate was determined as aforementioned. To examine the AIS *trans*-inhibition on AMPK and other AMPK-RKs, the catalytic activities of rat AMPK KD-UBA/AID (100 nM, towards 10 μM SAMS), human MELK KD-UBA (10 nM, towards 20 μM AMARA) and human MARK1 KD-UBA (500 nM, towards 100 μM AMARA) were determined in the absence or presence of SAD-A AIS-KA1 (50 μM). Each data set with increasing amounts of indicated inhibitory fragment was fitted to the non-competitive inhibition equation *v*_i_/*v*_0_=(*K*_i_+*a*[*I*])/(*K*_i_+[*I*]), where *K*_i_ and *a* are the apparent inhibition constant and residual activity, respectively.

### Crystallography

Crystals of mouse SAD-A KD-UBA were grown using the hanging-drop vapour diffusion technique by mixing the protein (∼10 mg ml^−1^) with an equal volume of reservoir buffer containing 0.1 M Tris, pH 8.0, 15% PEG MME 2000 at room temperature. The crystals were equilibrated in a cryoprotectant buffer containing reservoir buffer supplemented with 20% ethylene glycol before snap-frozen in liquid nitrogen.

The SAD-A KD-UBA were mixed with various C-terminal AIS-KA1 fragments in 1:1.5 molar ratio. Each mixture was incubated on ice for 1 h, and then purified by size exclusion chromatography. All complex proteins were subjected to crystallization trials, and crystals of the complex containing the AIS-KA1 fragment of residues 519–653 were obtained at 4 °C with a reservoir solution containing 0.1 M Na citrate, pH 5.2, 1.0 M Na malonate. Fresh crystals were quickly transferred to a cryoprotectant buffer containing 25% glycerol and flash-frozen under cold nitrogen stream at 100 K.

All diffraction data sets were collected at a wavelength of 0.98 Å and temperature of 100 K at beamline 17U at Shanghai Synchrotron Radiation Facility (SSRF, Shanghai, China) and processed using HKL2000 (ref. 64). The structure of mouse SAD-A KD-UBA were solved by molecular replacement using Phaser^65^ with MARK1 KD-UBA structure (PDB code: 2HAK) as search model. The complex structure of KD-UBA and AIS-KA1 was solved by molecular replacement using SAD-A KD-UBA and MARK1 KA1 domain (PDB code: 3OSE) as search models. Standard refinement was performed using Phenix^66^ and Coot^67^. The data processing and refinement statistics were summarized in Table 1. The structure validation was carried out using MolProbity^68^. All protein residues are in the favoured and allowed regions of the Ramachandran plot, and none are in disallowed regions. The clash score were 6.7 (94th percentile) for the KD-UBA structure and 5.16 (99th percentile) for the KD-UBA-AIS-KA1 structure, while the overall MolProbity score were 1.99 (74th percentile) and 2.02 (95th percentile), respectively. All structural representations in this paper were prepared with PyMOL (http://www.pymol.org).

### Observation of presynaptic puncta in *C. elegans* DA9 neuron

The 3 kb promoter sequence of *sad-1* (Psad-1) was amplified from *C. elegans* genome and cloned into pCFJ151 through *SpeI/XhoI* sites. The wild-type or mutant *sad-1* sequence was inserted into pCFJ151-Psad-1 through *XhoI* site. All constructs were verified by DNA sequencing.

The *C. elegans* strains (N2, EG4322 and wyIs85) were maintained, cultured and crossed using standard techniques^69^. The single-copy insertion of transgenes was performed as reported^70^. The wild-type or mutant *sad-1* plasmid at 50 ng μl^−1^ was injected to EG4322 with pJL43.1 (Pglh-2::transposase) at 50 ng μl^−1^, pGH8 (Prab-3::mCherry) at 10 ng μl^−1^, pCFJ90 (Pmyo-2::mCherry) at 2.5 ng μl^−1^ and pCFJ104 (Pmyo-3::mCherry) at 5 ng μl^−1^. Worms with normal locomotion and co-injection marker were picked. After starvation and chunked for three generation, worms that without fluorescence marker and move normally were isolated and validated by PCR. Animals were immobilized with 2,3-butanedione monoxamine (30 mg ml^−1^; Sigma-Aldrich), and images were collected with Olympus FV-1000 confocal microscope with an Olympus PlanApo × 40 objective at 2.5 zoom, a 488 nm Argon laser (GFP, green fluorescent protein).

In the rescue experiments, we crossed *sad-1* (wild-type or UBA/AIS mutants) transgenic worms with the protein-null mutant *sad-1*(*ky289*), and images of homozogote worms were taken using the Carl Zeiss fluorescence microscope at × 60.

### Lipid overlay assay

Phosphatidylcholine, phosphatidylethanolamine, phosphatidylglycerol, phosphatidylserine, sphingomyelin and cholesterol were purchased from Avanti Polar Lipids, phosphatidylinositol-4,5-bisphosphate (PIP_2_) was purchased from Echelon Biosciences, and phosphatidic acid was purchased from Larodan Fine Chemicals. The phospholipids were dissolved in methanol/chloroform/water (2:1:0.8, v/v/v), and 0.5 nmol of each phospholipid was spotted and air-dried on immobilon-NC membrane (Millipore). The membrane was blocked with 4% BSA for 1 h at room temperature, and then incubated with GST-tagged SAD-A proteins for 4 h at 4 °C. After extensive washing with PBS buffer, the bound proteins were detected by immunoblotting analysis with an anti-GST antibody (1:5,000; Sigma-Aldrich, G1160).

## Additional information

**How to cite this article:** Wu, J.-X. *et al*. Structural insight into the mechanism of synergistic autoinhibition of SAD kinases. *Nat. Commun.* 6:8953 doi: 10.1038/ncomms9953 (2015).

## Supplementary Material

Supplementary Information Supplementary Figures 1-8 and Supplementary Table 1

## Figures and Tables

**Table 1:** Data collection and refinement statistics for crystals.

	**KD-UBA**	**KD-UBA+AIS-KA1**
Data collection*		
Space group	*P1*	*C121*
Cell dimensions		
*a*, *b*, *c* (Å)	43.3, 60.7, 73.9	98.1, 87.3, 80.3
α, β, γ (°)	103.7, 106.5, 107.4	90, 92.3, 90
Resolution (Å)	30.00–2.00 (2.03–2.00)†	50.00–2.49 (2.58–2.49)
*R*_sym_ or *R*_merge_	8.7 (46.0)	8.7 (71.9)
*R*_p.i.m._ (%)	7.6 (38.8)	3.4 (27.8)
*I*/*σI*	8.2 (1.3)	26.9 (4.8)
CC_1/2_§	0.618	0.934
Completeness (%)	96.2 (85.7)	100.0 (100.0)
Redundancy	2.3 (1.9)	7.5 (7.5)
		
*Refinement*		
Resolution (Å)	29.68–2.00 (2.05–2.00)	34.58–2.49 (2.59–2.49)
No. reflections	41,937	23,731
*R*_work_/*R*_free_	19.0/22.9	19.7/23.6
No. atoms		
Protein	5,153	3,440
Ligand/ion	0	8
Water	163	67
*B*-factors		
Protein	48.6	54.5
Ligand/ion	0	63.7
Water	48.1	43.3
R.m.s. deviations		
Bond lengths (Å)	0.008	0.008
Bond angles (°)	1.110	1.085

AIS, autoinhibitory sequence; KA1, kinase associated; KD, kinase domain; r.m.s.,root mean squared; UBA, ubiquitin associated.

^*^Each data set was collected from a single crystal.

^†^Values in the parentheses are for highest resolution shell.

^§^Values are for highest resolution shell.
