# Supplementary material for: Structural insight into the mechanism of synergistic autoinhibition of SAD kinases
Source: Nat Commun. 2015 Dec 2;6:8953. doi: 10.1038/ncomms9953 (PMC4686854; doi:10.1038/ncomms9953)
Supplement: Supplementary Information — Supplementary Figures 1-8 and Supplementary Table 1 [file ncomms9953-s1.pdf]

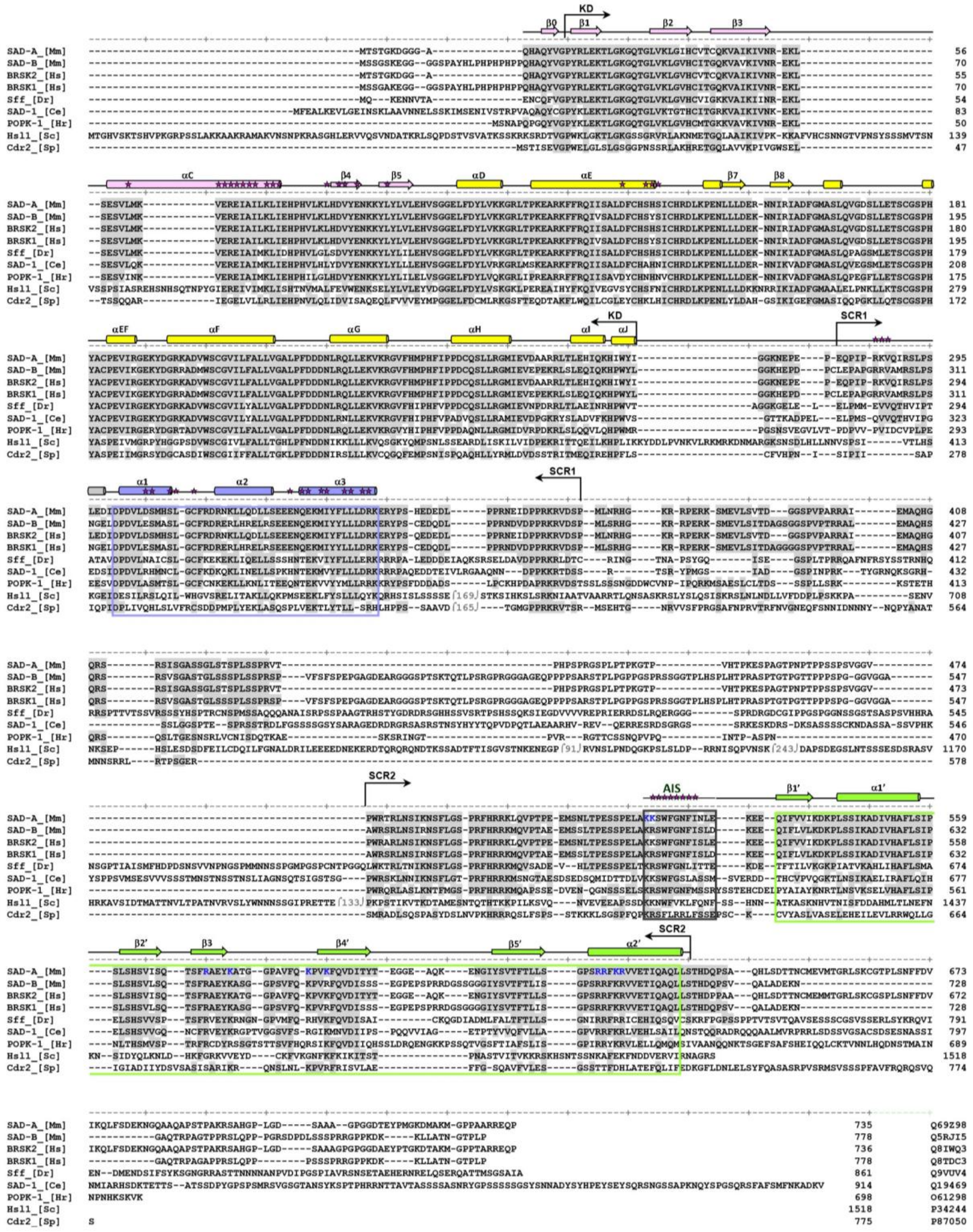

Supplementary Figure 1 | Sequence alignment of SAD kinases generated by ClustalW. The newly identified AIS sequence and the conserved UBA and KA1 domains within the non-catalytic region are boxed. Residues involved in the intramolecular interactions are indicated by asterisks, and those involved in lipid binding are highlighted in blue. *Mm, mouse; Hs, human; Dr, drosophila; Ce, C. elegans; Hr, H. roretzi; Sc, S. cerevisiae; Sp, S. pombe.*

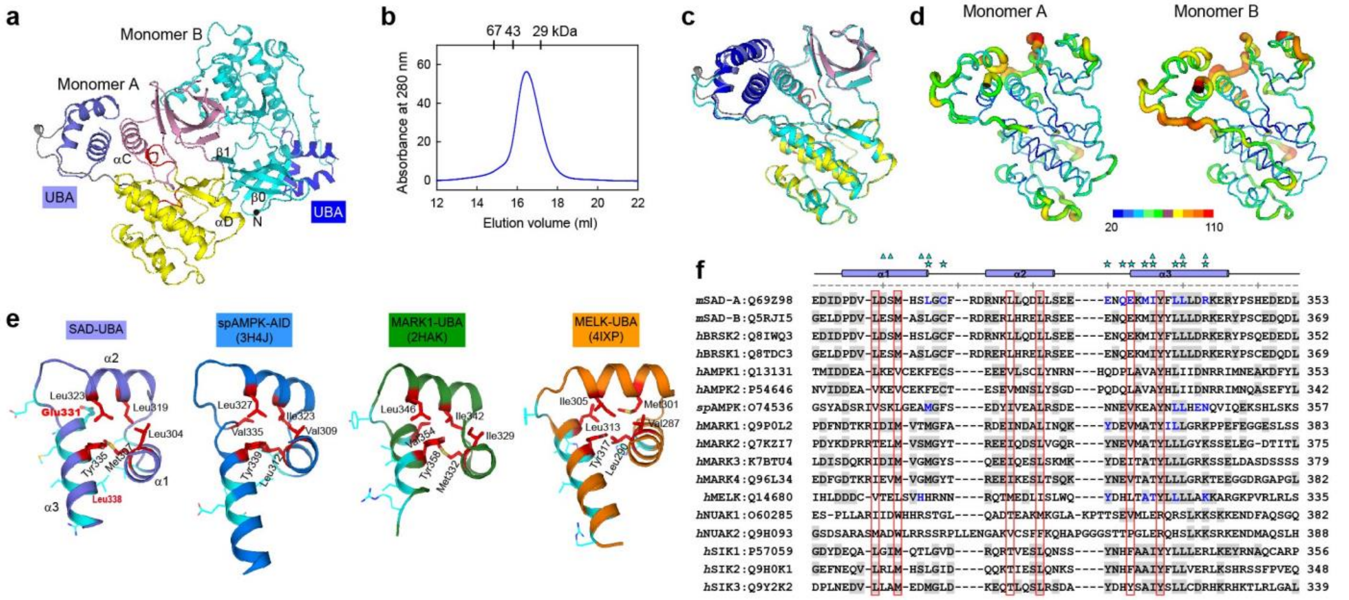

Supplementary Figure 2 | Comparison of the determined KD-UBA structures from AMPK family members. (**a**) Schematic representation of two molecules within one asymmetric unit of mouse SAD-A KD-UBA. Two molecules form a head-to-tail crystallographic dimer with helix αD of one monomer interacting with the N-terminal extension from the other monomer. (**b**) Determination of the monomeric nature of mouse SAD-A KD-UBA by gel filtration chromatograph. The retention time of KD-UBA (~50 μM) corresponds to an apparent molecular mass of 35~40 kDa, in reasonable agreement with its calculated monomeric molecular mass of 38 kDa. (**c**) Two molecules in the asymmetric unit adopt essentially the same conformation. Superposition of these two molecules yields an r.m.s. deviation of 0.3 Å among all the C_α atoms. (**d**) Comparison of Monomer A (left) and B (right) colored according to the temperature factors for C_α atoms. (**e**) Conserved conformation of the UBA domains from AMPK family members. Residues in the hydrophobic core are highlighted as red sticks, and those involved in interactions with kinase domain are corlored in cyan. Notably, Glu331 at the N-terminus of helix α3 in SAD-UBA is positioned differently from the corresponding residues of AMPK and other related kinases. Glu331 in SAD-A does not stabilize the UBA conformation, but rather forms salt bridge with Arg66 on the prominent αC helix. (**f**) Sequence comparison of the UBA domains from AMPK family members. Residues forming the hydrophobic core are boxed in red. The key residues of SAD-UBA involved in the interactions with kinase domain and AIS are indicated by asterisks and triangles, respectively. *m, mouse; h, human; sp, S. pombe.*

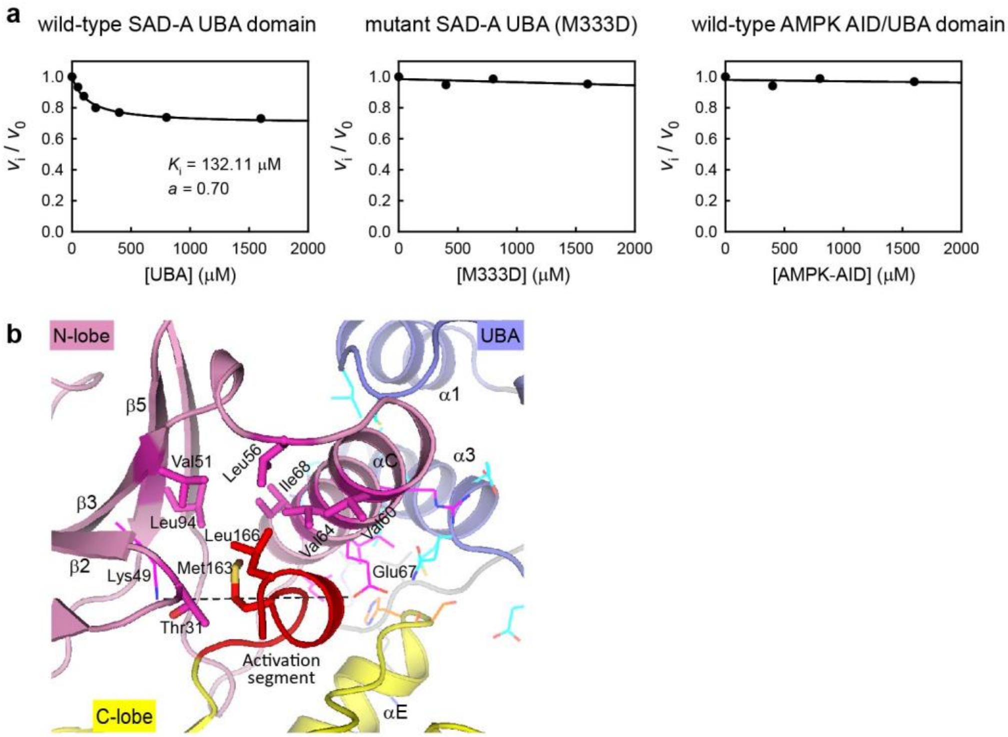

Supplementary Figure 3 | Structure and function of SAD-UBA. (**a**) *Trans*-inhibition of the UBA domain on the catalytic activity of SAD-A kinase domain. The assays were performed with 5 nM SAD-A kinase domain, 20 μM Cdc25C peptide and increasing amounts of the indicated UBA domain. Data were fitted to the non-competitive inhibition equation $v_i/v_0 = (K_i+a[I])/(K_i+[I])$, where K_i and a are the apparent inhibition constant and residual activity, respectively. The continuous curve was derived using this equation and the best-fit parameters were indicated. (**b**) Close-up view of the active site of SAD-A kinase domain. Notably, Met163 and Leu166 from the activation segment penetrate into the hydrophobic pocket formed by Thr31 from the Gly-rich loop, Val51 on strand β3, Leu56 from the β3-αC loop, Val60, Val64, Ile68 on helix αC and Leu94 on strand β5.

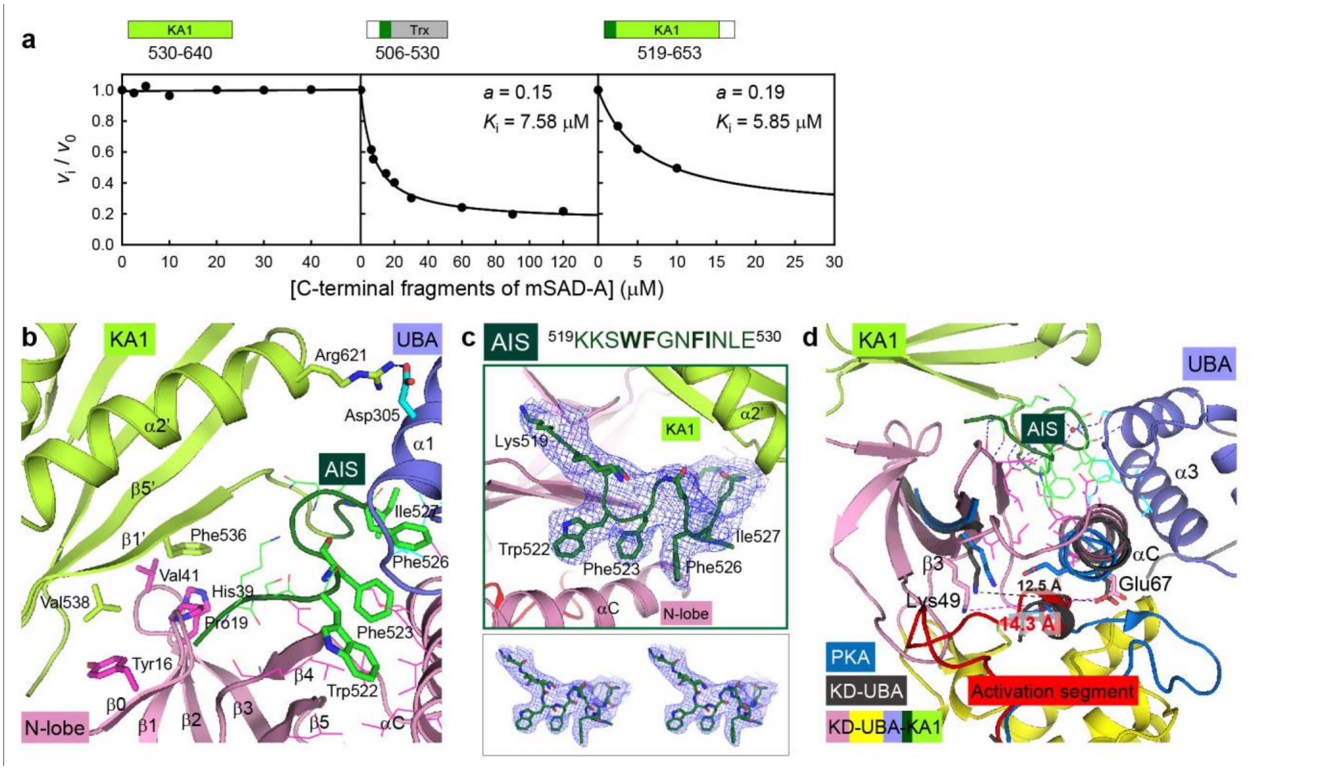

Supplementary Figure 4 | AIS, rather than KA1, inhibits the kinase activity of SAD. (**a**) *Trans*-inhibition of different C-terminal fragments on the activity of the KD-UBA fragment (10 nM). The data sets were fitted to the non-competitive inhibition equation as that in Figure 4a and Supplementary Figure 3a. (**b**) The weak interaction between the KA1 domain and the KD-UBA fragment. Residues Phe536 and Val538 from strand β1' of the KA1 domain make weak hydrophobic contacts with residues Tyr16, Pro19, His39 and Val41 from the kinase N-lobe β-sheet, and Arg621 on KA1 α2' helix forms a salt bridge with Asp305 on UBA α1 helix. (**c**) The F_o-F_c omit map (contoured at 2.0 σ) clearly shows electron density for the AIS sequence. For clarity, the UBA domain is omitted. Shown at the bottom is the stereo image of the F_o–F_c omit map for the AIS peptide. (**d**) Comparison of the active site conformations in the structures of the KD-UBA and AIS-KA1 complex, the KD-UBA alone, and PKA (PDB code: 1ATP). For clarity, only the strands β3, helices αC and activation segments of the isolated KD-UBA (dark grey) and PKA (marine blue) are displayed. The highly conserved residues, Lys on strand β3 and Glu on helix αC, are highlighted in sticks, and the distances between Lys49 and Glu67 in two SAD-A structures are indicated. Residues involved in interactions between the KD-UBA and AIS-KA1 fragments of SAD-A are shown as lines.

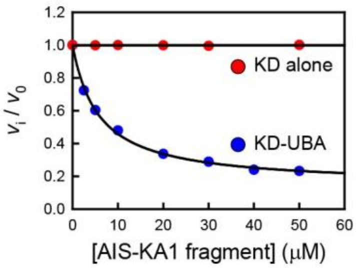

Supplementary Figure 5 | Comparison of the AIS *trans*-inhibition on the activities of the kinase domain alone and the KD-UBA fragment. The *trans*-inhibition assays were performed with 10 nM enzyme (the kinase domain alone or the KD-UBA fragment) and various concentration of the AIS-KA1 fragment. The AIS-KA1 fragment effectively inhibited the activity of the KD-UBA fragment, but not the kinase domain alone, clearly demonstrating that the inhibitory effect of AIS on SAD activity depends on the UBA domain.

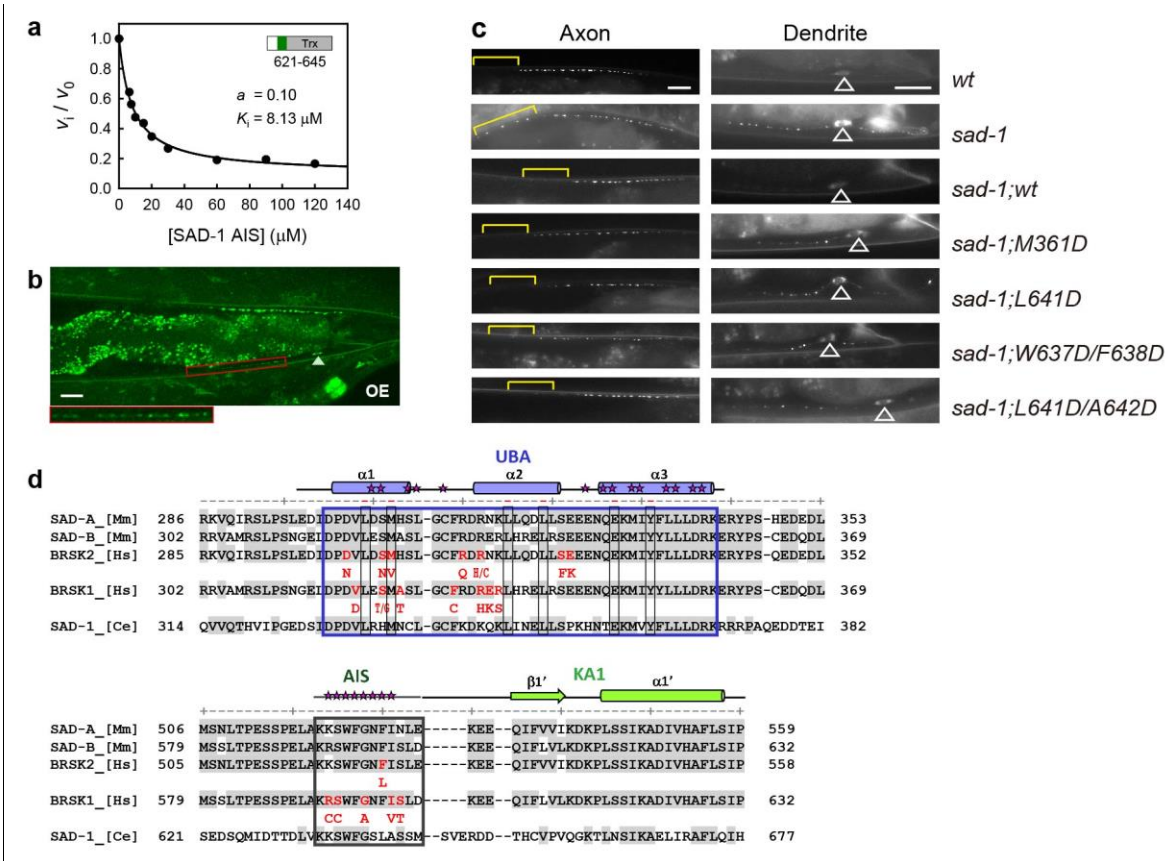

Supplementary Figure 6 | Conserved autoinhibition in *C. elegans*. (**a**) *Trans*-inhibition of the corresponding AIS sequence of *C. elegans* ortholog SAD-1 on the activity of mouse SAD-A KD-UBA. The assay was performed with 10 nM SAD-A KD-UBA and increasing amounts of SAD-1 AIS. The result revealed that the *C. elegans* AIS sequence had evident inhibitory role on the activity of mouse SAD-A. (**b**) Localization of synaptic vesicle-associated GFP::RAB-3 in *sad-1* overexpressing animals. Scale bar, 10 μm. (**c**) Axonal and dendritic defects of *sad-1*(*ky289*) protein-null animals rescued by wildtype and mutant *sad-1*. The wildtype *sad-1* rescued both synaptic organization at the distal end of DA9's axon and neuronal polarity in the dendrite region; however, the UBA and AIS mutants restored synaptic organization but not neuronal polarity. Scale bar, 10 μm. (**d**) SNPs reported in the UBA domains and the AIS sequences of human BRSK kinases. Seven UBA residues (D301N, S305N, M306V, R313Q, R315H/C, S324F and E325K) and one AIS residue (F525L) of SAD-A/BRSK2 and seven UBA residues (V319D, S322T/G, A324T, F329C, R332H, E333K and R334S) and five AIS residues (R593C, S594C, G597A, I600V and S601T) of SAD-B/BRSK1 are altered and highlighted in red. The key interacting residues are indicated by asterisks, and residues forming the hydrophobic core of UBA are boxed in black. *Mm, mouse; Hs, human; Ce, C. elegans.*

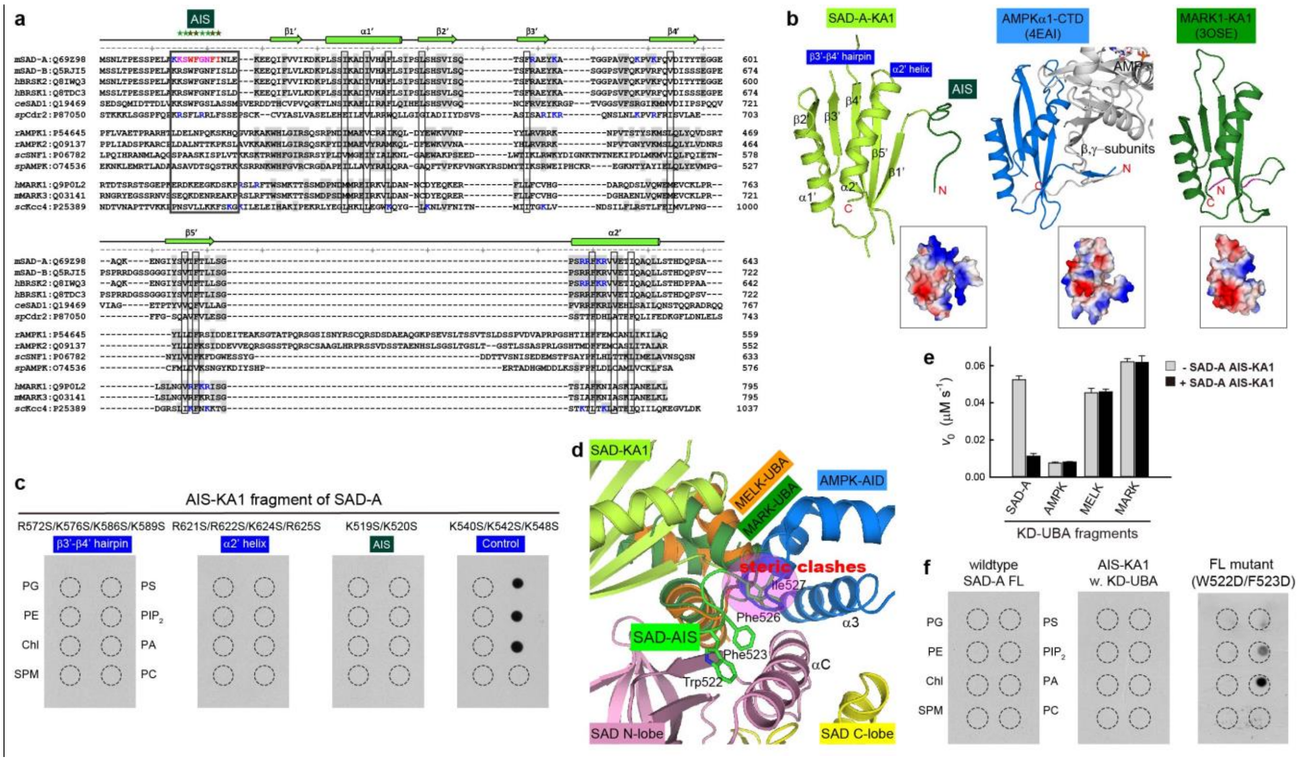

Supplementary Figure 7 | The AIS-KA1 fragment mediates lipid/membrane association of SAD-A. (**a**) Sequence alignment of the AIS-KA1 fragments from SAD/BRSK, AMPK and MARK kinases. The positively charged residues that are required for phospholipid binding of SAD-A, Cdr2, MARK1 and Kcc4 are highlighted in blue, and the SAD-AIS residues interacting with KD-UBA are indicated. *h, human; m, mouse; r, rat; ce, C. elegans; sp, S. pombe; sc, S. cerevisia.* (**b**) Comparison of the KA1 domains from SAD, MARK and AMPK. Shown at the bottom of each KA1 is the corresponding surface representation colored according to electrostatic potential (positive, blue; negative, red). The C-terminal domain of AMPK α-subunit is structurally conserved to the KA1 domains of SAD and MARK, but it functions differently to mediate the assembly of AMPK heterotrimer. This compact conformation is stabilized by multiple van der Waals interactions involving highly conserved hydrophobic residues indicated in panel a. (**c**) Protein-lipid overlay assays for the AIS-KA1 mutants. (**d**) Steric clashes of the AIS bound to the SAD-A KD-UBA with the UBA domains from AMPK (3H4J), MARK1 (2HAK) and MELK (4IXP) upon superposition of kinase N-lobes. For clarity, the SAD-UBA is omitted, and only the UBA domains from AMPK, MARK1 and MELK are displayed. (**e**) *Trans*-inhibition of SAD-A AIS-KA1 on the KD-UBA activities of AMPK and other AMPK-RKs. (**f**) Protein-lipid overlay assays for the full-length SAD-A (wildtype and mutant W522D/F523D) and the complex of KD-UBA and AIS-KA1.

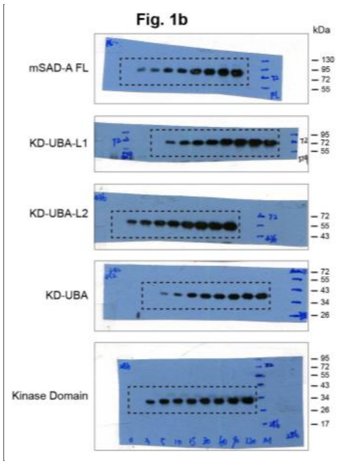

Supplementary Figure 8 | Uncropped images of blots in Figure 1b. Cropped regions are indicated with rectangles as appropriate.

Supplementary Table 1 | Kinetic parameters for SAD-A mutants

mSAD-A		k_{cat} (s^{-1})	$K_{m(Cdc25C)}$ (μM)	k_{cat} / $K_{m(Cdc25C)}$ (μM^{-1} s^{-1})
pKD		48.60 ± 0.75	12.06 ± 0.53	4.03 ± 0.24
pKD-UBA		16.45 ± 0.23	10.23 ± 0.43	1.61 ± 0.09
pKD-UBA mutants	K287A	16.36 ± 0.26	9.25 ± 0.45	1.76 ± 0.11
	L310D	20.08 ± 0.72	13.05 ± 1.54	1.53 ± 0.24
	C312A	17.26 ± 0.58	12.06 ± 1.51	1.58 ± 0.22
	E328A	41.35 ± 1.62	10.23 ± 0.43	3.43 ± 0.56
	Q330A	45.36 ± 1.00	11.57 ± 0.90	3.92 ± 0.39
	E331K	43.56 ± 1.26	10.91 ± 1.13	3.99 ± 0.53
	M333D	35.72 ± 1.00	10.55 ± 0.99	3.39 ± 0.41
	I334D	44.96 ± 0.98	10.87 ± 0.85	4.13 ± 0.41
	L337D	36.87 ± 0.95	10.26 ± 0.97	3.59 ± 0.43
	R341A	20.24 ± 0.63	11.48 ± 1.25	1.76 ± 0.25
